# Proton beam and carbon ion radiotherapy in skull base chordoma: a systematic review, meta-analysis and meta-regression with trial sequential analysis

**DOI:** 10.1007/s10143-024-03117-1

**Published:** 2024-12-07

**Authors:** Amanda Cyntia Lima Fonseca Rodrigues, Salem M. Tos, Ahmed Shaaban, Georgios Mantziaris, Daniel M. Trifiletti, Jason Sheehan

**Affiliations:** 1 Department of Neurology, University of North Carolina at Chapel Hill, Chapel Hill, NC USA; 2 Department of Neurological Surgery, University of Virginia, Charlottesville, VA USA; 3 Department of Radiation Oncology, Mayo Clinic Florida, Jacksonville, FL USA

**Keywords:** Skull base chordoma, Proton, Carbon ion, Radiotherapy, Meta-analysis

## Abstract

Chordomas are rare, locally aggressive tumors that have a high rate of recurrence, especially at the skull base. This systematic review and meta-analysis aimed to analyze the efficacy and safety of proton beam therapy (PBT) and carbon ion radiotherapy (CIRT) for skull base chordoma. We conducted a systematic search of MEDLINE, EMBASE, CENTRAL, Web of Science, and Ovid up to November 26, 2023, following the PRISMA statement. Studies involving more than 10 patients with skull base chordoma treated with PBT or CIRT were included. The outcomes analyzed were local control, overall survival, progression-free survival, and toxicities. Meta-analysis was performed using the Mantel–Haenszel method and the inverse variance method. Fourteen studies met the inclusion criteria, encompassing 1,145 patients (671 treated with PBT and 474 with CIRT). No significant difference was found between PBT and CIRT for 5-year local control (LC) and overall survival (OS). The only timepoint with a difference in local control was at 3 years, when PBT showed an advantage in local control (90% vs. 83% for CIRT; *p* = 0.05) and progression-free survival was similar (94% for PBT vs. 83% for CIRT; *p* = 0.09). Sensitivity analysis and meta-regression revealed no significant influence of predefined parameters on outcomes. Publication bias was suggested by asymmetrical funnel plots. Both PBT and CIRT are effective treatments for skull base chordoma, with comparable long-term efficacy. This meta-analysis underscores the need for individualized treatment approaches and further research to refine these therapies in clinical practice.

**Supplementary Information:**

The online version contains supplementary material available at 10.1007/s10143-024-03117-1.

## Introduction

With an incidence of 1/1000000 and 0.2% of all intracranial tumors, chordoma is a rare locally aggressive tumor [1, 2]. As they arise from notochord remnants, their location is usually in the midline mainly sacral (29.2%–45%) or clivus (26–32%) or spine (23–32.8%) [1, 3]. According to WHO they are classified into three subtypes: conventional, dedifferentiated and poorly differentiated chordoma (PDC) [4]. Management of skull base chordoma is based on a multi-disciplinary approach due to the high rate of recurrence (50%), and difficulty to achieve gross total resection (GTR) due to proximity to critical structures.

Even when GTR is achieved, recurrence-free survival at 10 years is only 31% [2, 3, 5, 6]. Adjuvant therapy includes radiotherapy, either photon- or particle-based [7]. Chordoma is considered relatively radioresistant so higher than usual radiation doses are required for local control which may adversely impact surrounding critical structures such as the cranial nerves or the brainstem [8]. Therefore, particle therapy (proton or carbon ion therapy) is often utilized for patients with recurrent or residual chordomas as it can achieve conformal high radiation doses and less extension to surrounding structures [7]. We aim through this systematic review and meta-analysis to investigate the efficacy and safety of proton and carbon ion therapy for skull base chordoma.

## Materials and methods

### Search strategy

This study was conducted using Preferred Reporting Items for Systematic Reviews and Meta-Analyses (PRISMA) statement [9]. We systematically searched electronic databases, including MEDLINE, EMBASE, CENTRAL, Web of Science, and Ovid from inception to November 26, 2023. The review protocol was registered at PROSPERO (CRD42022375433). Ethical approval and patient consent were not required because data was synthesized from previously published studies. The search strategy can be found in Supplementary Material 1.

### Eligibility criteria

The eligibility criteria included prospective and retrospective observational studies of more than 10 patients diagnosed with skull base chordoma based on pathological assessment. These studies involved patients who received particle therapy using proton beam therapy (PBT) or carbon ion radiotherapy (CIRT). The studies should report at least one of the outcomes of interest, which includes incidence of toxicity, and survival outcomes, including local control (LC), overall survival (OS), progression-free survival (PFS), and toxicities. The incidence of toxicity was defined as both acute (occurring within 90 days of treatment) and late (occurring after 90 days), graded according to the Common Terminology Criteria for Adverse Events (CTCAE). For survival analyses, OS and PFS were calculated from the time of initial diagnosis for de novo cases and from the time of salvage treatment for recurrent cases. Studies were excluded if they involved non-human subjects, were not in English, used overlapping data from the same institution(s), and had a follow-up duration of less than 1 year. Additionally, studies on patients receiving other RT techniques, including photons, brachytherapy, and other particles, as well as studies including only reviews, case reports, abstracts, letters to the editor, and editorials were also excluded.

### Study selection and data extraction

The study selection process involved screening titles, abstract, and full-text articles. Three independent authors (ACLFR, SMT, AS) conducted these screening according to inclusion and exclusion criteria, and any conflicts that arose were resolved through discussion and mutual consensus. The opinion of an author (GM) was also requested as needed to arbitrate any disparities or uncertainties. A standardized excel sheet was used for data extraction and tabulation. The extracted variables included demographic and patient characteristics, and outcomes of interest.

### Quality of assessment

Quality assessment of the risk of bias (Supplementary Material 1) was performed using the Risk Of Bias in Non-Randomized Studies of Interventions tool (ROBINS-I) [10]. The papers and their characteristics were classified into: low; moderate; serious, and critical risk of bias. Three independent reviewers (ACLFR, SMT and AS) assessed the risk for bias. When there was disagreement, it was resolved by discussion among reviewers.

### Statistical analysis

The meta-analysis was performed using R version 4.3.2 (R Core Team (2021). R: A language and environment for statistical computing. R Foundation for Statistical Computing, Vienna, Austria. URL https://www.R-project.org/.) with the packages ‘meta’ and ‘metafor [11]. The pooled relative risk was estimated using the Mantel–Haenszel method for comparison between proton and carbon, and the inverse variance for single-arm analysis with subgroup comparisons was performed indirectly through single-arm meta-analysis, comparing PBT and CIRT [12]. Significance values for these subgroup comparisons were calculated, depicting differences in outcomes such as LC, OS and PFS [13]. Forest plots were generated to display results. We assessed the publication bias using Egger’s test for funnel plot asymmetry with the trim and fill method [14]. Influence diagnostics were used to detect the studies which influenced the overall estimate of our meta-analysis the most and let us assess if this large influence distorts our pooled effect using four influence diagnostic plots: a Baujat plot, influence diagnostics and sensitivity analysis using leave-one-out meta-analysis results, sorted by effect size and I^2^ value for each outcome [15]. Meta-regression was conducted using continuous variables, including GTV and total radiation dose, to assess their potential influence on the outcomes [16]. Trial sequential analysis (TSA) was performed for 5-year LC and OS. The required information size (RIS) was evaluated by setting a relative risk reduction of 20%, the first type of error (α = 0.05) and power of 80%. The conclusions were statistically significant when the cumulative Z-curve crossed the TSA boundary [17].

## Results

### Eligible studies

**Fig. 1:**
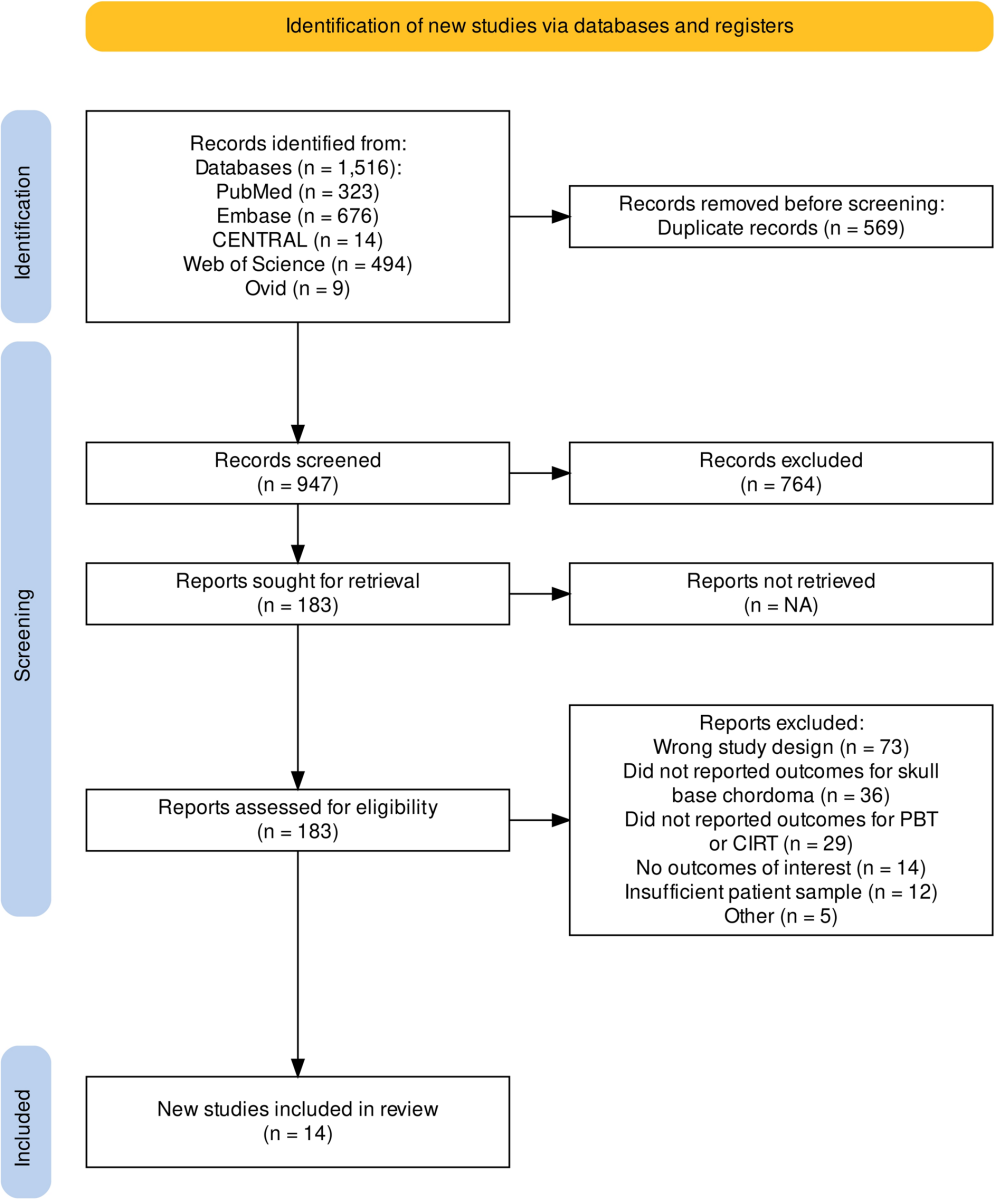
PRISMA Flow Diagram

The search yielded a total of 1,516 publications. After 569 duplicate studies were detected and eliminated, we further removed 764 articles by titles and abstract screening. Subsequently, 183 articles were considered for full-text screening. Finally, 14 studies that met inclusion criteria published from 2009 to 2023 were included in this systematic review [2, 7, 18–29]. The PRISMA flow diagram (Fig. 1) illustrates the study selection process.

### Study and patient characteristics

Of the fourteen included studies, ten were retrospective cohorts, three were prospective cohorts, and one was a clinical trial. Patients were treated between 1981 and 2020 at a diverse array of institutions throughout the world in which four studies were conducted in North America (USA), three in Asia (Japan), and the remaining seven were conducted in Europe (Switzerland, Germany, UK, Italy).

A total of 1,145 patients with skull base chordoma were included, 671 treated with PBT (58.6%) and 474 with CIRT (41.4%). The median age ranged from 13 to 58 years (range: 11 – 92 years). Twelve studies with 907 patients specified the sex of patients; 497 patients were male (54.8%) with a ratio of male to female of 1.21. The median follow-up after radiation therapy varied from 21 to 108 months. Data including study characteristics, patient demographics, tumor features and details about radiation type and regimen for each study are presented in Table 1. A summary of both acute and late radiation-induced effects, detailing the frequency of occurrences and the corresponding toxicity grading levels based on the CTCAE, is provided in Table 2.

**Table 1:** Characteristics of the included studies with patient demographic

Study	Country	Study design	Quality of evidence	Particle therapy	Sample size	Age^c^ median (range)	Gender M/F	Previous surgery	Previous CTX	Tumor status^e^	Histology	Target volume median (range)	Treatment regimens median (range)		BC	OPC	Follow-up^h^ median (range)
													Total dose	Dose per fraction			
Ares 2009	Switzerland	R	Level 2	PBT	42	< 20 = 3 20 ≤ 40 = 12 40 ≤ 60 = 17 > 60 = 10	18/24	–	NR	Primary 33 Recurrence 9	–	–	73.5 CGE (67–74)	1.8–2 Gy (RBE)	18	–	34 (14–92)
Chhabra 2023	USA	P	Level 2	PBT	61	58 (18–92)	55/45	Upfront surgical resection	NR	Recurrence 3	–	–	74 Gy (RBE) (21–86)	1.8 Gy (RBE) (1.2–2)	–	–	22 (3–286)
Deraniyagala 2014	USA	R	Level 2	PBT	33	–	26/7	GTR 9 STR 22 Biopsy 2	NR	All recurrence	–	–	74 CGE (70–79)	–	11	3	21 (3–58)
Gaito 2023	UK	P	Level 2	PBT	177	13 (1.0–81)	–	–	NR	–	–	–	54 Gy (RBE)	–	–	–	40 (1–139)
Hayashi 2016	Japan	R	Level 3	PBT	19	52 (13–76)	8/11	STR 8 Partial 9 Biopsy 2 All OS	NR	All recurrence	–	–	77.44 Gy^f^ 78.4 Gy^g^	1.2 Gy^f^ 1.4 Gy^g^	–	–	61.7 (31.5–115.4)
Hong 2023	USA	R	Level 3	PBPT 22 (69%) PSPT 10 (31%)	32	51 ± 17^d^	15/17	GTR 22 STR 10 EEA 23 EEA + OC fusion 3 MicroEA 1 ETC 1 OS + EEA 2 OS 2	NR	All residual	–	–	PBPT 72 ± 3^d^ PSPT 74 ± 3^d^	PBPT 2 PSPT 1.9	–	–	59 (35–86)
Iannalfi 2020	Italy	P	Level 2	PBT CIRT	70^a^ 65^b^	53 (17–81)^a^ 58 (13–81)^b^	40/30^a^ 42/23^b^	GTR 19^a^ 0^b^ Partial 51^a^ 64^b^ Biopsy 0^a^ 1^b^ EEA 57^a^ 55^b^ Transcranial 8^a^ 5^b^ Prior surgeries 1 = 43^a^ 1 = 34^b^ > 1 = 27^a^ > 1 = 31^b^	NR	Primary 61^a^ 46^b^ Recurrence 9^a^ 19^b^	–	–	74 (72–74)^a^ 70.4 (70.4-70.4)^b^	2 Gy^a^ CIRT 3 Gy^b^	PBT 17 CIRT 14	PBT 64 CIRT 58	49 (6–87)
Koto 2020	Japan	R	Level 2	CIRT	34	52 (16–76)	16/18	–	NR	Recurrence 7 Residual tumor 22 Naive tumor 5	–	GTV 18.7 cm^3^ (1.5–126.7)	60.8 Gy (RBE)	3.8 Gy	–	–	108 (9–175)
Mattke 2023	Germany	R	Level 2	PBT CIRT	36^a^ 111^b^	50^a^ 51^b^	22/14^a^ 63/48^b^	–	NR	Primary 28^a^ 85^b^ Recurrence 8^a^ 26^b^	–	CTV 38.3ml^a^ 40.9ml^b^	74 Gy (RBE)^a^ 66 Gy (RBE)^b^	PBT 2 Gy CIRT 3 Gy	56	–	36.5^a^ 52.2^b^
McDonald 2016	USA	R	Level 2	PBT	39	52 (17–78)	21/18	Prior surgeries 1 = 27 2 = 10 3 = 2	NR	–	Conventional 31 (79.5%) Chondroid 8 (20.5%)	GTV 8.1 cm^3^ (0–79.7)	77.4 Gy (70.2–79.2)	–	9	–	51 (2–106)
Schulz-Ertner 2007	Germany	CT	Level 2	CIRT	96	47 (11–80)	54/42	–	NR	Primary 59 Recurrence 37	–	PTV2 80.3 ml (13.9–594.2)	60 CGE (60–70)	3 CGE	–	–	31 (3–91)
Takagi 2018	Japan	R	Level 2	PBT CIRT	11^a^ 13^b^	55.5 (24–79)	10/14	–	NR	Primary 17 Recurrence 7	–	GTV 17.0 cm^3^ (0.4–113.1)	65 Gy	2.5 Gy	–	–	71.5 (14–175)
Uhl 2014	Germany	R	Level 2	CIRT	155	48 (15–85)	76/79	GTR 85 Biopsy 16	NR	Primary 101 Recurrence 54	–	PTV2 60 ml (54–70) PTV1 45 ml (45–52.8)	60 Gy	3 Gy	–	–	72 (12–165)
Weber 2016	Switzerland	R	Level 3	PBT	151	43.3 ± 18.3^d^	86/65	GTR 4 STR 146 All OS Prior surgeries 1 = 63 2 = 59 3 = 14 4 = 6 5 = 3	NR	Recurrence 36	–	GTV 35.4 cm^3^ (27.5)^d^	72.5 ± 2.2 Gy_RBE_	1.8–2 Gy	17	38	50 (4–176)

*R* retrospective, *P* prospective, *CT* clinical trial, *PBT* proton beam therapy, *PBPT* pencil beam proton therapy, *PSPT* passive scatter proton therapy, *CIRT* carbon ion radiotherapy, *CTX* chemotherapy, *GTR* gross total resection, *STR* subtotal resection, *OS* open surgery, *EEA* endoscopic endonasal approach, *OC* occipito-cervical, *MicroEA* microscopic endonasal, ETC endoscopic high transcervical, *NR* not reported, *GTV* gross tumor volume, *CTV* clinical target volume, *PTV2* boost planning target volume, *PTV1* initial planning target volume, *CGE* cobalt gray equivalent, *Gy* gray, *RBE* relative biological effectiveness, *BC* brainstem compression, *OPC* optic pathway compression

^a^PBT group

^b^CIRT group

^c^Time in years

^d^Mean ± SD

^e^At the time of radiotherapy

^f^in 9 patients

^g^in 10 patients

^h^Time in months

**Table 2:** Description of toxicities according to the Common Terminology Criteria for Adverse Events (CTCAE)

Study	Time	Toxicity description
Ares 2009	Acute	Asymptomatic circumscribed white-matter changes in the temporal lobe (Grade 1 leukoencephalopathy) (5), occurred at 10, 18, 31, 35, and 48 months after PT. In four patients, the MRI changes remained stable or resolved spontaneously. In one patient, who developed MRI changes at 18 months, MRI findings progressed up to the time of this analysis. However, she continues to remain asymptomatic
	Late	Grade 3 unilateral optic neuropathy (1), 20 months after treatment. Grade 3 symptomatic temporal lobe parenchyma damage (2) at 12 and 19 months after treatment. Grade 4 unilateral optic-neuropathy (1), 12 months after treatment
Chhabra 2023		Toxicities of radiation not specific for skull base chordoma
Deraniyagala 2014		Grade 2 Unilateral hearing loss (6), partially corrected by a hearing aid
Gaito 2023		Toxicities undeferentiated from chondrosarcoma
Hayashi 2016	Acute	None of the 19 patients experienced acute reactions over grade 2
	Late	Mild cognitive and memory dysfunction (1) 3 years after the end of PBT. MR images demonstrated bilateral medial temporal lobe radiation necrosis (RN). mild neurological symptoms (2) with slight high-intensity changes on T2 weighted MRI in the medulla. Numbness in the left leg (1), 9.5 months after the end of PBT. Slight weakness of left extremities associated with numbness (1), 13 months after the end of PBT
Hong 2023	Acute	Acute toxicities (nausea, fatigue, skin irritation, alopecia) were observed in 3 cases with PSPT and 7 cases with PBPT, all graded CTCAE 1–2
	Late	Non-acute toxicities in PSPT included endocrine (7), ophthalmic (1), otologic (3), radiation necrosis (2), and other toxicity (1), while PBPT had endocrine (4), ophthalmic (2), otologic (4), radiation necrosis (2), and other (4). Among 30 non-acute toxicities, 20 were CTCAE 1–2, 30% were grade 3, and one case was not applicable (radiation-induced meningioma)
Iannalfi 2020	Acute	There were no observed acute toxicities of high grade (Grade ≥3)
	Late	Specific toxicities were observed in the following: ear (8 cases, including 7 with Grade 3 and 1 with Grade 4), endocrine (1 case with Grade 3), eye (4 cases, with 2 having Grade 3 and 2 having Grade 4), and nervous system disorders (3 cases, all with Grade 3)
Koto 2020	Acute	Dermatitis cases were predominantly Grade 0 (20), followed by Grade 1 (14), with no occurrences of higher-grade toxicity. Mucositis during this phase exhibited a majority of Grade 0 cases (15), with decreasing numbers for Grades 1 to 3 (11, 7, and 1)
	Late	Specific adverse events included brain stem injury, brain injury, mucositis, bone fracture, optic nerve injury, hearing impairment, and hypoglossal nerve disorder. In the majority of cases, late-phase events were Grade 0, indicating no or mild toxicity. Instances of Grade 1 or 2 were present, with rare occurrences of Grade 3 or 4 and no reports of Grade 5 events
Mattke 2023	Acute	The observed effects were similar between carbon ions and protons, with acute side effects such as mucositis or skin toxicity generally being mild (none exceeding grade 2)
	Late	Among 44 patients with temporal lobe reactions, the most severe late side effect was necrosis, observed in 20 patients. The majority were asymptomatic (CTC grade 1) or responded well to therapy with steroids or bevacizumab (CTC grade 2-3)
McDonald 2016		Nausea (4) or mucositis with weight loss (4), severe headache, and visual deterioration (1), radiation necrosis (2), sensorineural hearing loss (4), chronic mastoid effusions (4), and pituitary dysfunction (2). Grade 1: 28 Grade 2: 17 Grade 3: 2 Grade 4: 2
Schulz-Ertner 2007	Acute	18 cases experienced focal hair loss with accompanying Grade 1 skin erythema in 5 cases. Temporary middle ear issues were reported in 11 cases. Mucositis occurred in 36 cases (Grade 1), 5 cases (Grade 2), and 2 cases (Grade 3). Nausea (Grade 1) was reported in 3 cases, conjunctivitis (Grade 1) in 1 case, and diplopia aggravation (Grade 2) was observed in 3 cases during radiation therapy
	Late	Grade 3 optic nerve neuropathy in four cases. Three of these patients, with preexisting unilateral visual field deficits from tumor involvement, developed unilateral blindness after carbon ion therapy. Additionally, a 69-year-old woman experienced bilateral blindness
Takagi 2018	Acute	Dermatitis was observed in 8 cases (Grade 1) and 3 cases (Grade 2). Similarly, mucositis was documented with 5 cases (Grade 1) and 2 cases (Grade 2). No patient experienced ≥ Grade 3. regarding late reaction after either PBT or CIT
	Late	Toxicities featured Grade 2 occurrences in brain necrosis, optic nerve disorder, nerve system disorders, hearing impairment, middle ear inflammation, and pharyngeal hemorrhage. Additionally, Grade 3 late toxicity was observed for optic nerve disorder and nerve system disorders, while Grade 4 was reported for brain necrosis and pharyngeal hemorrhage. PBT group: six patients encountered late toxicities of Grade 2 or higher, with four cases classified as Grade 2 and two as Grade 3. Similarly, in CIT, six patients experienced late toxicities of Grade 2 or higher, including four cases of Grade 2, one case of Grade 3, and one case of Grade 4
Uhl 2014		NA
Weber 2016		Unilateral optic neuropathy (5), grade 4 bilateral optic neuropathy (2), grade 3 temporal lobe necrosis (13), cerebellum brain parenchyma, grade 3 necrosis (1), grade 4 spinal cord necrosis (1), and grade 3 unilateral hearing loss (3)

### Local tumor control and overall survival with trial sequential analysis

**Fig. 2:**
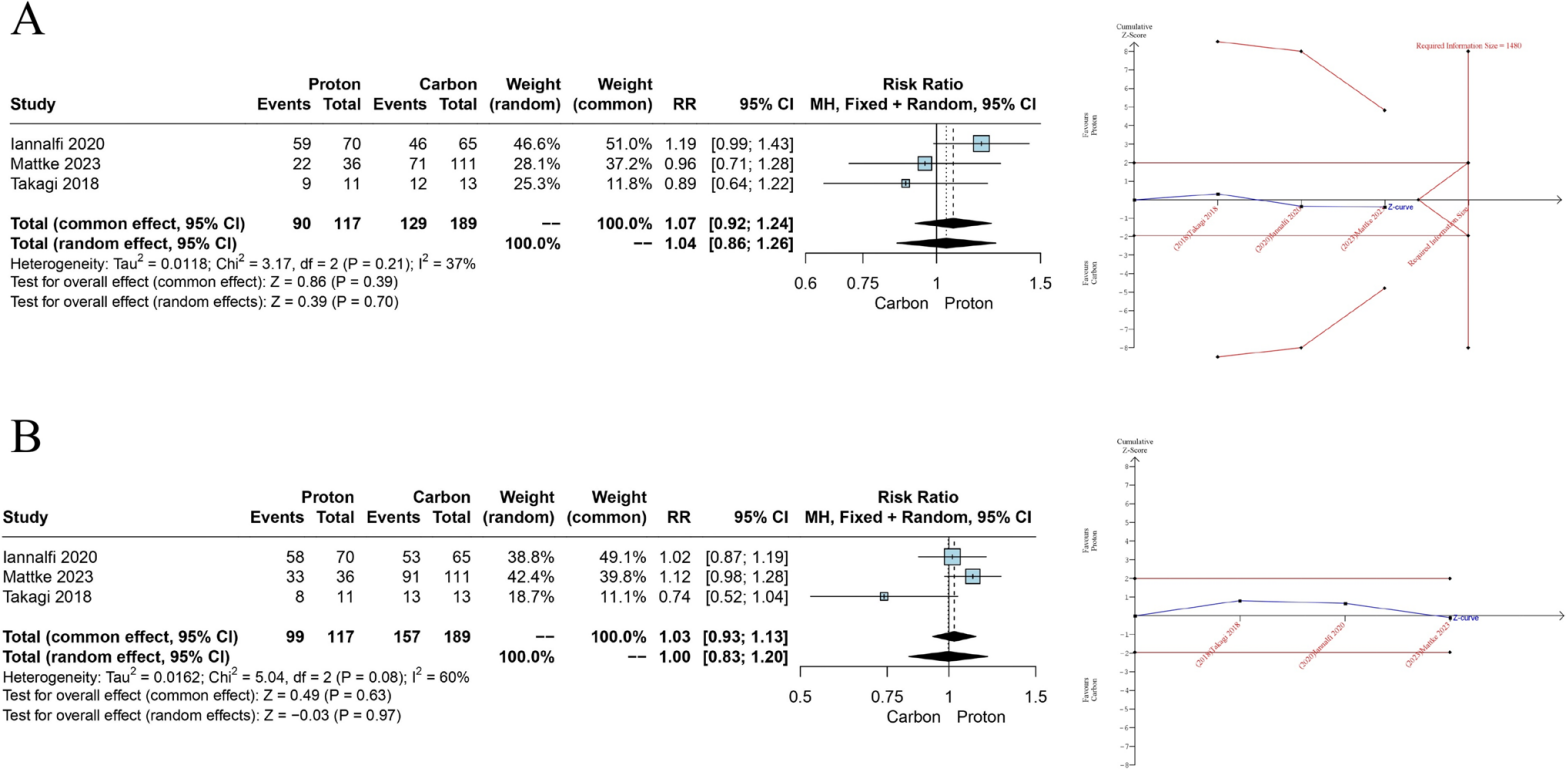
**A** Meta-analysis and TSA results of 5-year LC, **B** Meta-analysis and TSA results of 5-year OS

All three studies that directly compare PBT with CIRT, reported 5-year LC and OS [7, 23, 27]. No significant difference between the two therapies for the 5-year LC (RR = 1.04, 95% CI = 0.86 to 1.26, *p* = 0.70, Fig. 2A) and the 5-year OS (RR = 1.00, 95% CI = 0.83 to 1.20, *p* = 0.97, Fig. 2B) was detected.

TSA of PBT versus CIRT for 5-year LC and OS showed that the information size has not been reached, indicating a lack of evidence and the meta-analysis result is inconclusive with the cumulative Z-curve crossing neither the conventional significance threshold nor the O’Brien-Fleming boundaries (Fig. 2A, B). More studies are needed to confidently conclude on potential effects of PBT on 5-year LC and OS.

### Local tumor control

**Fig. 3:**
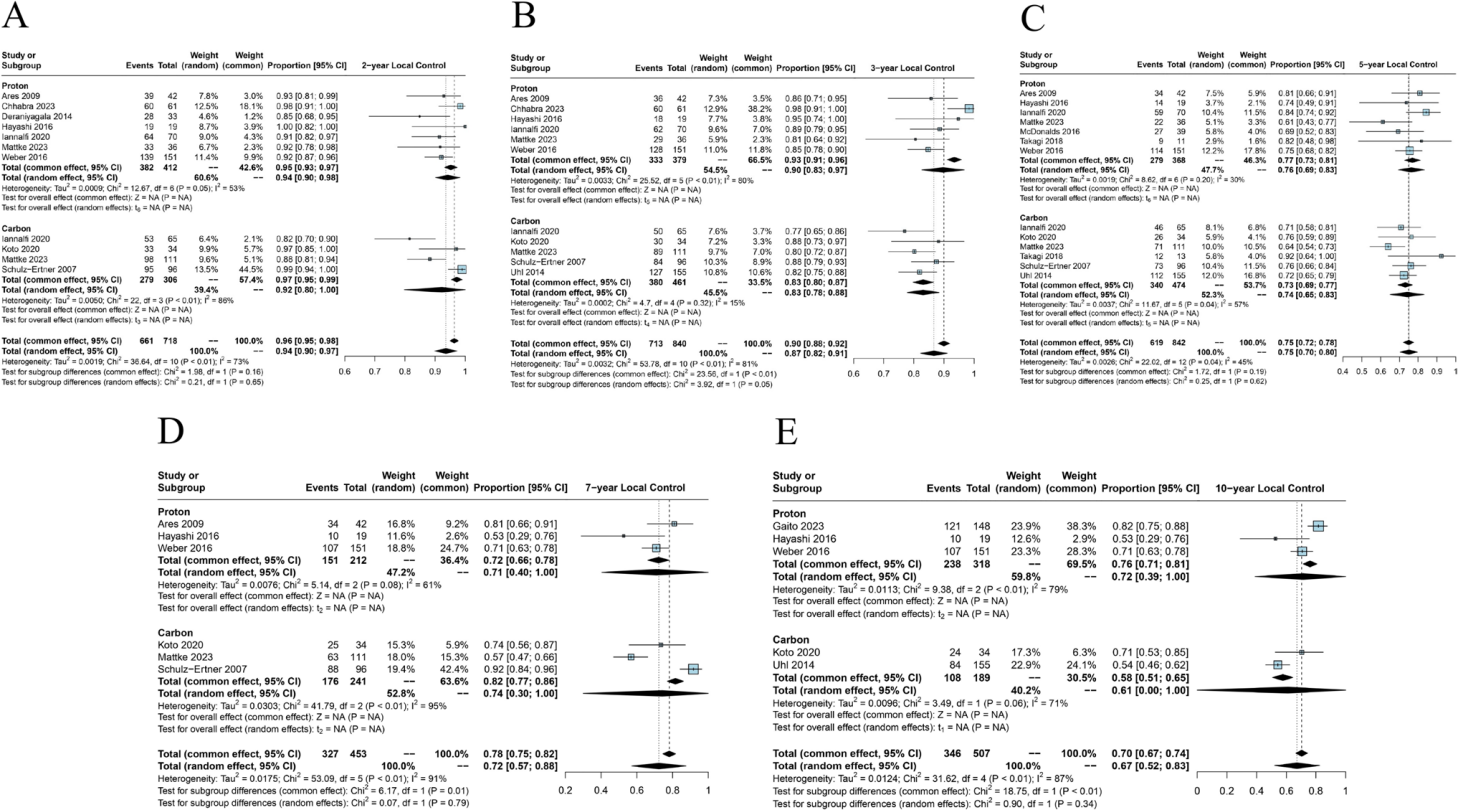
**A** Forest plot depicting meta-analysis for 2-year LC, **B** Forest plot depicting meta-analysis for 3-year LC, **C** Forest plot depicting meta-analysis for 5-year LC, **D** Forest plot depicting meta-analysis for 7-year LC, **E** Forest plot depicting meta-analysis for 10-year LC

Thirteen studies included information about LC [2, 7, 18–21, 23–29]. The indirect analysis of PBT and CIRT for LC was performed at various time points (2-, 3-, 5-, 7-, and 10-year). At 3-year, PBT exhibited a LC of 90% (95% CI = 0.83 to 0.97), compared to 83% (95% CI = 0.78 to 0.88) with CIRT; the subgroup difference test was (*p* = 0.05) (Fig. 3B). The pooled LC rates were calculated at 2-year [PBT 94% (95% CI = 0.90 to 0.98) vs CIRT 92% (95% CI = 0.80 to 1.00), Fig. 3A], 5-year [PBT 76% (95% CI = 0.69 to 0.83) vs CIRT 74% (95% CI = 0.65 to 0.83), Fig. 3C], 7-year [PBT 71% (95% CI = 0.40 to 1.00) vs CIRT 74% (95% CI = 0.30 to 1.00), Fig. 3D], and 10-year [PBT 72% (95% CI = 0.39 to 1.00) vs CIRT 61% (95% CI = 0.00 to 1.00), Fig. 3E], respectively. The tests for subgroup differences indicated no significant variation at these time points.

### Overall survival

**Fig. 4:**
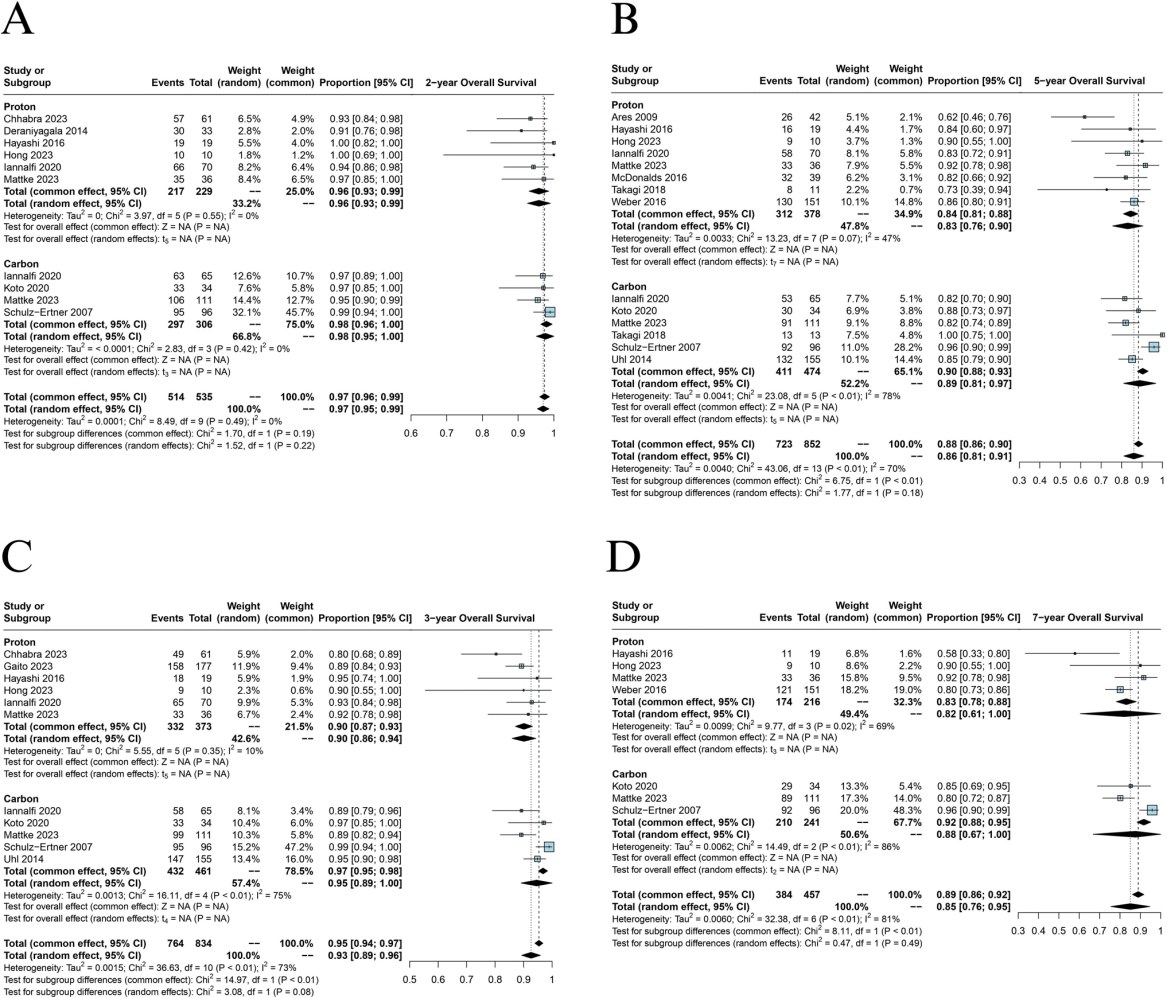
**A** Forest plot depicting meta-analysis for 2-year OS, **B** Forest plot depicting meta-analysis for 3-year OS, **C** Forest plot depicting meta-analysis for 5-year OS, **D** Forest plot depicting meta-analysis for 7-year OS

All fourteen studies evaluated OS [2, 7, 18–29]. The pooled OS rates were calculated at 2-year [PBT 90% (95% CI = 0.86 to 0.94) vs CIRT 95% (95% CI = 0.89 to 1.00), Fig. 4A], 3-year [PBT 83% (95% CI = 0.76 to 0.90) vs CIRT 89% (95% CI = 0.81 to 0.97), Fig. 4B], 5-year [PBT 83% (95% CI = 0.76 to 0.90) vs CIRT 89% (95% CI = 0.81 to 0.97), Fig. 4C], and 7-year [PBT 82% (95% CI = 0.61 to 1.00) and CIRT 88% (95% CI = 0.67 to 1.00), Fig. 4D], respectively. The tests for subgroup differences had no significant effect on the variation of these time points.

### Progression-free survival

**Fig. 5:**
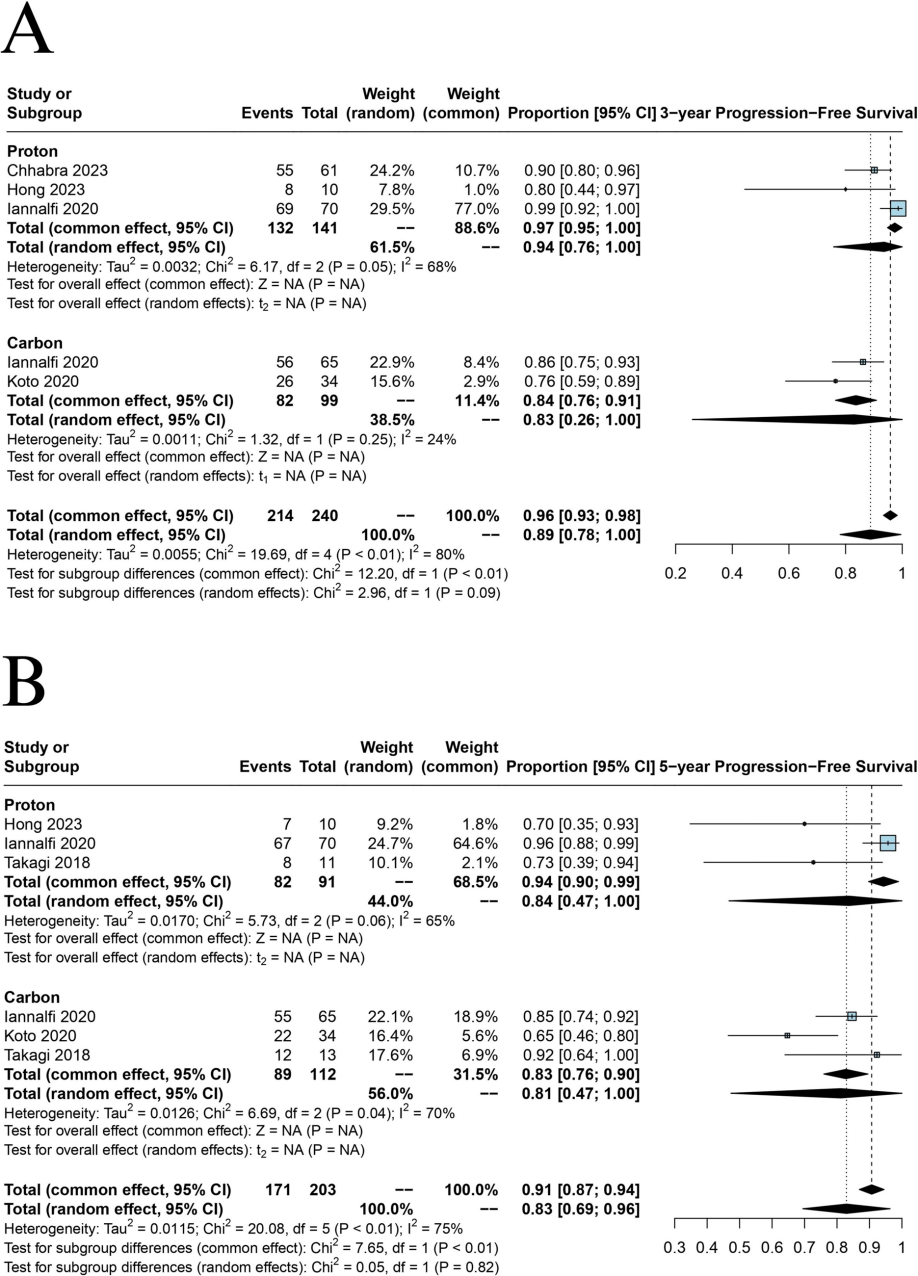
**A** Forest plot depicting meta-analysis for 3-year PFS, **B** Forest plot depicting meta-analysis for 5-year PFS

Five studies analyzed PFS at 3- and 5-year [2, 22–24, 29]. At 3-year, PBT showed a PFS of 94% (95% CI = 0.76 to 1.00), while CIRT had 83% (95% CI = 0.26 to 1.00); the subgroup difference test was (*p* = 0.09) (Fig. 5A). At 5-year [PBT 84% (95% CI = 0.47 to 1.00) vs CIRT 81% (95% CI = 0.47 to 1.00), Fig. 5B] both therapies demonstrated comparable PFS with no significant subgroup differences.

### Toxicity

**Fig. 6:**
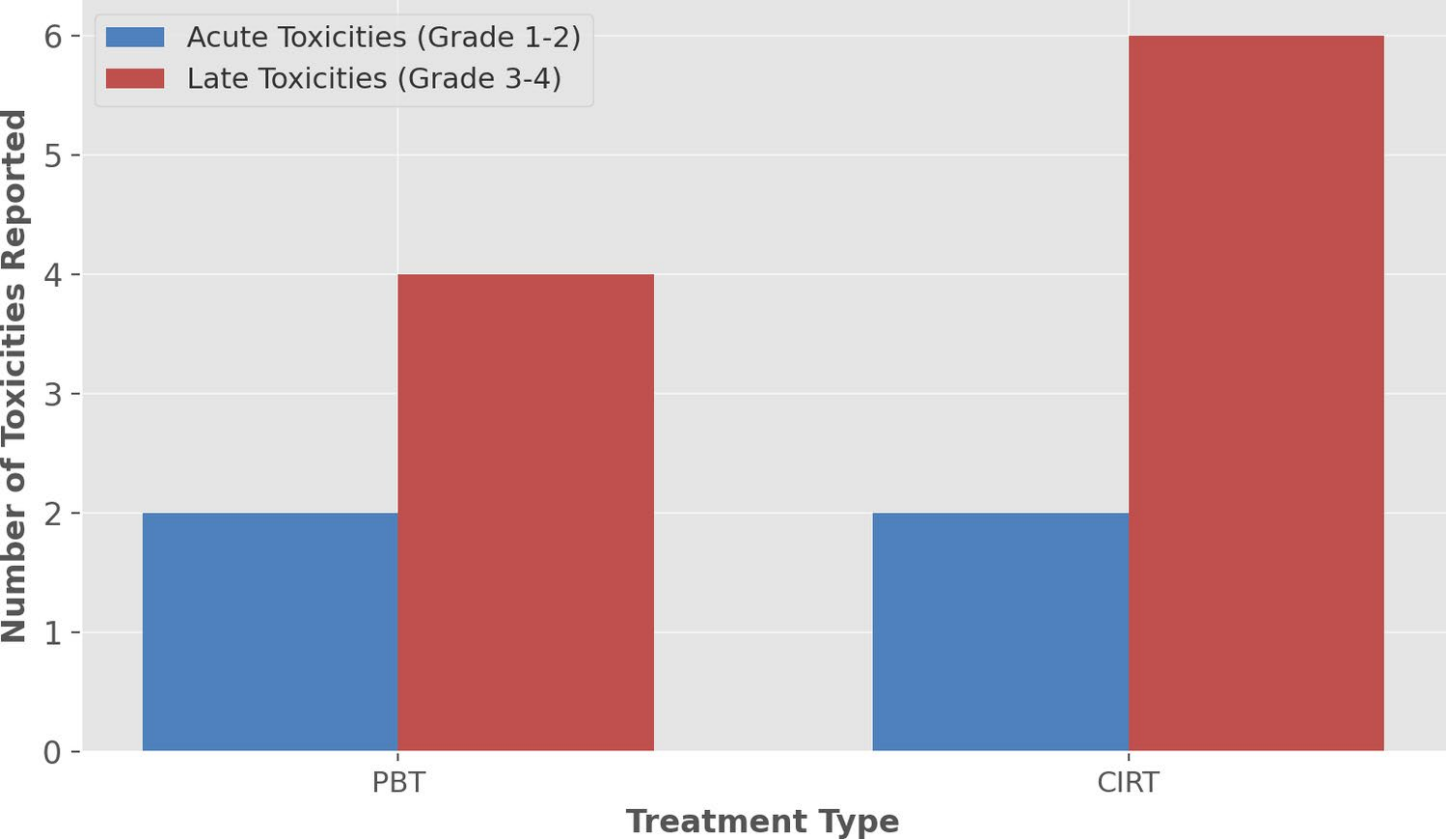
Bar plot displaying acute toxicities (grade 1–2) and late toxicities (grade 3–4) for proton beam therapy and carbon ion radiotherapy

A range of acute and late toxicities was observed for both PBT and CIRT. Acute toxicities, such as mild dermatitis, mucositis, and nausea, were generally low grade (mostly grades 1 and 2) across both treatments. No occurrences of higher-grade acute toxicities were reported. Late toxicities were more severe, including cases of optic neuropathy, brain necrosis, and hearing loss. Grade 3 and 4 toxicities were more frequently associated with CIRT, particularly radiation necrosis (Fig. 6).

### Single survival curves

**Fig. 7:**
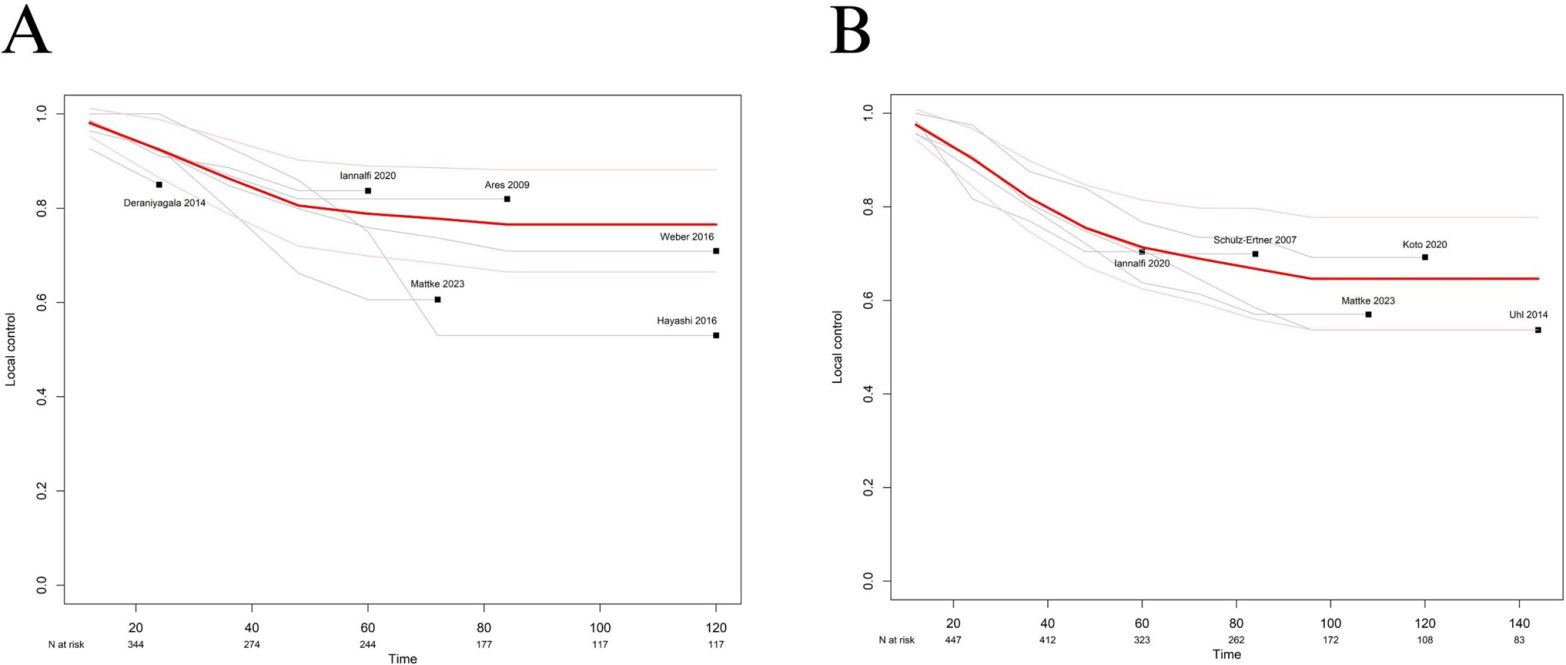
Pooled summary local control of **A** proton beam therapy, **B** carbon ion therapy

Individual patient data (IPD) were extracted for LC from the included studies to obtain reconstructed survival curves. The pooled 1-, 3- and 5-year LC of PBT were 94.8% (95% CI = –90.2 to 99.6), 83.7% (95% CI = 69.4 to 92.4), and 78.4% (95% CI = 88.5) (Fig. 7A). The pooled 1-, 3- and 5-year LC of CIRT were 94.1% (95% CI = –90 to 98.7), 80.2% (95% CI = –72.9 to 88.3), and 71.6% (95% CI = 62.7 to 81.6) (Fig. 7B).

### Sensitivity analysis

Sensitivity analysis was conducted to see the influential studies and the heterogeneity, the plots were sorted by the effect size, I^2^ heterogeneity and Baujat of all the outcomes using the leave-one-out method. No single study was identified as a remarkably influential study and removing any single study altered neither outcomes nor heterogeneity remarkably (Supplementary Material 2).

### Publication bias

The funnel plot distribution of scattered points in the outcomes analyzed was asymmetrical, suggesting that there was a possibility of publication bias, which could be related to the heterogeneity between studies (Supplementary Material 3).

### Meta-regression

For the 5-year LC, meta-regressions showed no impact to significantly predict outcome on covariates GTV (estimate 0.01; SE 0.01; 95% CI − 0.00 to 0.02; Z value 1.59; *p* = 0.11), and radiation dose (estimate 0.05; SE 0.03; 95% CI − 0.01 to 0.10; Z value − 1.63; *p* = 0.10) (Supplementary Material 1, Supplementary Material 4A).

At 5-year OS the covariates GTV (estimate − 0.01; SE 0.01; 95% CI − 0.01 to 0.03; Z value − 1.29; *p* = 0.20), and radiation dose (estimate 0.05; SE 0.04; 95% CI − 0.03 to 0.13; Z value − 1.17; *p* = 0.24) indicated no significant effect (Supplementary Table 2, Supplementary Material 4B).

## Discussion

This meta-analysis pooled the results of fourteen studies with 1,145 patients to compare the outcomes of PBT and CIRT in treating skull base chordomas. Our results reveal no significant difference in 3- and 5-year LC and OS between PBT and CIRT. While previous meta-analyses by Lu et al. [30] and Dong et al. [31] demonstrated comparable efficacy between these modalities, we incorporated TSA, which allows for a more rigorous evaluation of the cumulative evidence by determining whether the information size is sufficient to reach reliable conclusions, adding a new analysis by addressing the risk of random error, which was not explored in prior reviews. The meta-regression analysis examined continuous variables such as GTV and total radiation dose, enabling a more nuanced understanding of how these factors influence outcomes. Indirect comparisons of PBT and CIRT were employed through single-arm meta-analysis.

Proton therapy’s physical properties explain its efficacy and safety for skull base chordoma [18, 32]. In brief, proton particles pass through collimators with kinetic energy resulting in a conformal target. Based on Bragg peak principle, they have a sharp fall of the dose at the end and less side effects compared to photon therapy [32]. The European Society of Medical Oncology (ESMO) recommends doses of at least 74 GyE with conventional fractionation (RBE) (relative biological effectiveness) [33]. The reported local control rate with surgery followed by proton therapy alone ranges from 75.8% at 5 years to 70.9% at 7-year by Weber et al. [29]. Chhabra et al. [2] found 2 and 3-year local control of 97% and 94% respectively without high-grade toxicity in a large series including 61 skull base chordoma. Hong et al. [22] compared pencil beam proton therapy versus passive scattering proton therapy for clival chordoma without significant differences in outcome or toxicity. Factors reported to affect recurrence include histology (poorly differentiated), residual tumor volume, genetic mutation, proximity to the brain stem or optic apparatus (results in under dosage to avoid injury), and location in lower/middle clivus [22, 23, 29, 34–37]. As skull base chordoma rarely metastasizes, their local control is an independent favorable prognostic factor related to overall survival as reported by Fung et al. [38]. In addition, the same group reported gross total volume (GTV) > 25 cm^3^ as an unfavorable prognostic factor.

Even though carbon ions share properties with protons since they are both charged particles, they have higher linear energy transfer and deliver higher RBE selectively [23, 39]. Both proton therapy and carbon ions can achieve a steep dose gradient to surrounding critical structures [23, 40]. In current practice, CIRT is used in case of unfavorable tumor characteristics that may bias this cohort toward a more unfavorable outcome23. Uhl et al. [28] showed that a planned target volume below 75 ml achieved better local control and that 5-year and 10-year control of 72% and 54% respectively. Iannalfi et al. [23] showed target volumes < 10.4 cm^3^ were better treated with proton therapy, while larger volumes responded better to carbon ion therapy. Koto et al. [24] reported the 5- and 9-year local control rates as 76.9% and 69.2%, respectively. In the same series, GTV > 34.7 cm^3^ resulted in local control drop from 85.7% at 9 years to 26.7%. Mattke et al. [7] recently compared proton therapy to carbon therapy and reported 5-year local control of 61% and 65%, respectively. The prospective study by Iannalfi et al. [23] found a 5-year local control for proton therapy and carbon therapy of 84% and 71%, respectively.

Based on the previous series, no significant difference in toxicity rates is noted between the two modalities [7, 23]. Rates of asymptomatic radiotoxicity approaching 28% were reported for CIRT by Koto et al. [24], while Iannalfi et al. [23] reported 12% high-grade toxicity for both treatment modalities. Alahmari et al. [32] in a systematic review concluded that the toxicity of proton therapy is underestimated. Based on previous reports, temporal lobe necrosis is the most severe late complication [38, 41]. Other reports showed high-grade toxicity of 6–8.1% for proton therapy and 4.1–6% for carbon therapy [18, 26, 29, 40]. Further studies are needed to evaluate the safety of both modalities. Our findings into the toxicities associated with these treatments, reveal a spectrum of acute to late effects ranging in severity with most being low grade ones. Ares et al. [18] reported Grade 1 leukoencephalopathy in 5 patients post-PBT, with most cases stabilizing or resolving spontaneously. Grade 3 and 4 toxicities, such as unilateral optic neuropathy and symptomatic temporal lobe damage, were also observed. Chhabra et al. [2] and Deraniyagala et al. [19] contribute to our understanding of the differentiated toxicity profiles, including hearing loss, mild cognitive dysfunctions, and more severe outcomes like optic neuropathy and radiation necrosis.

GTV and proximity to critical structures were identified as independent predictors of local failure [23]. Similarly, the proximity of the tumor to the brainstem significantly impacted LC in patients with primary tumors, indicating that anatomical constraints may compromise target coverage and affect outcomes [7]. Improved outcomes were also observed in patients who underwent surgery before radiotherapy, highlighting the importance of surgical intervention in optimizing LC and OS [27].

### Limitations

Most studies are retrospective resulting in various practice protocols, and patient referral patterns. Also, chordomas are slowly growing tumors so long-term follow-up is required to assess treatment efficacy which was lacking in many studies. It is important to note that while a statistically significant difference in local control was observed at 3 years, this result may be due to chance, as no consistent differences were seen at other time points. This suggests the potential influence of random variability, and further studies with larger sample sizes are needed to clarify this finding. Finally, while our analysis is comprehensive, encompassing a substantial number of patients, the relative rarity of skull base chordomas limits the availability of large-scale, multicentric studies, which would provide more robust evidence.

## Conclusion

The current study highlights the safety and effectiveness of PBT and CIRT in treating skull base chordomas, emphasizing the need for personalized treatment based on patient and tumor specifics. Both modalities show comparable long-term efficacy and further work in each is underway to optimize delivery as its availability increases. This work supports the ongoing optimization of patient care and underscores the need for further research with larger cohorts to refine these treatments in clinical practice.

## Supplementary Information

Below is the link to the electronic supplementary material.

Supplementary file1 (DOCX 673 KB)

Supplementary file2 (DOCX 32.8 KB)

Supplementary file3 (DOCX 92.0 KB)

Supplementary file4 (DOCX 27.2 KB)

## Data Availability

No datasets were generated or analysed during the current study.

## Abbreviations

CENTRAL: Cochrane Central Register of Controlled Trials
CIRT: Carbon ion therapy
CTCAE: Common terminology criteria for adverse events
LC: Local control
PBT: Proton beam therapy
PDC: Poorly differentiated chordoma
PFS: Progression-free survival
PRISMA: Preferred reporting items for systematic reviews and meta-analysis
PROSPERO: International prospective register of systematic reviews
OS: Overall survival
RCT: Randomized controlled trial
RIS: Required information size
ROBINS-I: Risk of bias in non-randomized studies—of interventions
TSA: Trial sequential analysis
WHO: World Health Organization
