# Supplementary material for: Proton beam and carbon ion radiotherapy in skull base chordoma: a systematic review, meta-analysis and meta-regression with trial sequential analysis
Source: Neurosurg Rev. 2024 Dec 7;47(1):893. doi: 10.1007/s10143-024-03117-1 (PMC11625079; doi:10.1007/s10143-024-03117-1)

**Supplementary Material 1.** Summary plot and traffic-light plots presenting the quality assessment of included observational using the Risk Of Bias In Non-randomised Studies - of Interventions (ROBINS-I)

**Figure 1.** Summary plot


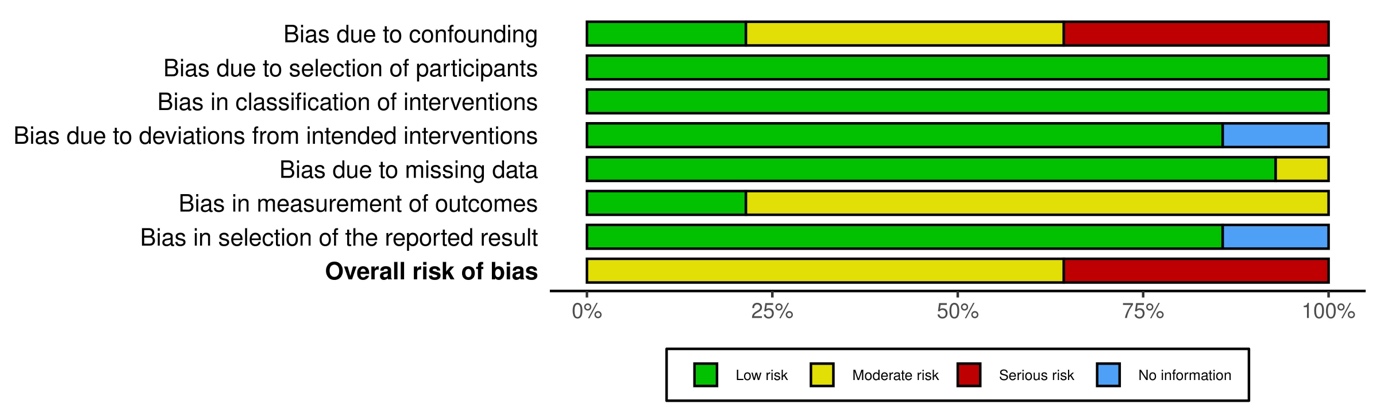


**Figure 2.** Traffic-light plot


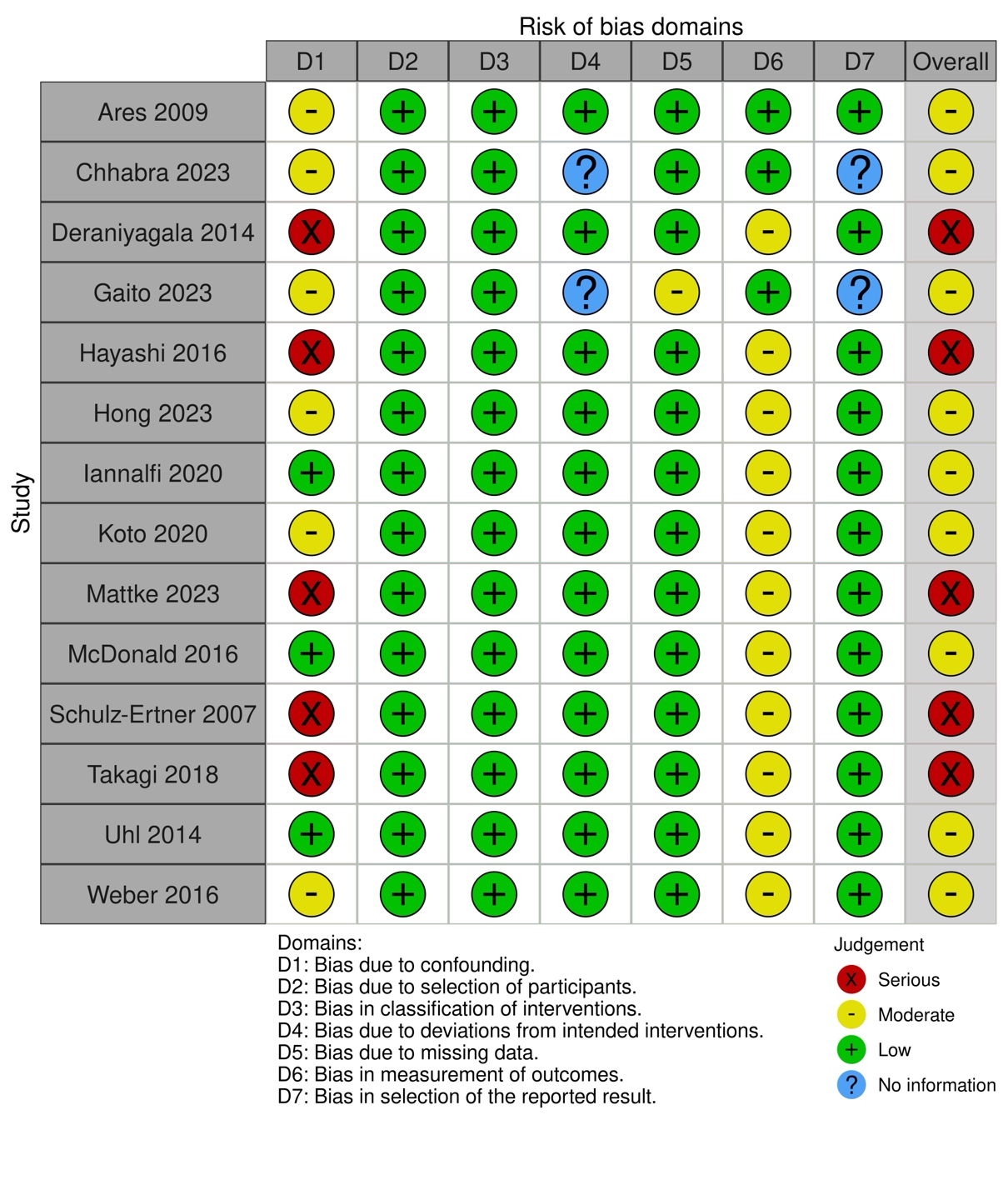

Supplement: Supplementary file 1 — Supplementary file1 (DOCX 673 KB) [file 10143_2024_3117_MOESM1_ESM.docx]
