# Supplementary material for: Proton beam and carbon ion radiotherapy in skull base chordoma: a systematic review, meta-analysis and meta-regression with trial sequential analysis
Source: Neurosurg Rev. 2024 Dec 7;47(1):893. doi: 10.1007/s10143-024-03117-1 (PMC11625079; doi:10.1007/s10143-024-03117-1)
Supplement: Supplementary file 3 — Supplementary file3 (DOCX 92.0 KB) [file 10143_2024_3117_MOESM3_ESM.docx]

**Supplementary Material 3.** Funnel plots showing publication bias

A

B
