# Supplementary material for: Proton beam and carbon ion radiotherapy in skull base chordoma: a systematic review, meta-analysis and meta-regression with trial sequential analysis
Source: Neurosurg Rev. 2024 Dec 7;47(1):893. doi: 10.1007/s10143-024-03117-1 (PMC11625079; doi:10.1007/s10143-024-03117-1)
Supplement: Supplementary file 4 — Supplementary file4 (DOCX 27.2 KB) [file 10143_2024_3117_MOESM4_ESM.docx]

**Supplementary Material 4.** Meta-regression bubble plots

A

B
